# Coffee in the Turkish Fashion

**Published:** 1858-01

**Authors:** 


COFFEE IN THE TURKISH FASHION.—Commonly, at least in Europe and in this country, the French method of preparing coffee for a beverage is deemed the best. Travellers in the East assure us, however, that coffee *á la Turque* is preferable to all, although at first it is not relished as much as when made in the common way. A late French traveller in Greece gives his vote in favor of the Oriental side of the question, and, at the same time, directions for preparing the Turkish coffee. We translate the former for the benefit of our untravelled readers, who may wish to make the trial of a little variety in their favorite morning beverage.

Roast, but without burning, the fresh or green berries; then reduce them to an impalpable powder, either in a mortar or in a suitable mill. Water is to be put on the fire and allowed to remain until it boils, when it is taken off, and to every measure of a coffeecupful there is to be added a small (tea) spoonful of coffee and the same quantity of powdered sugar, which are to be carefully stirred up together. The coffee-pot is again put on the fire until it boils up; it is then taken off, and once more replaced on the fire, after which it is removed, and the coffee now prepared is to be quickly poured into cups for immediate use. Some amateurs have it boiled five times; but three is deemed sufficient by the best judges. A skillful server will take care that each cup shall receive its share of the foam or bead which is formed on the surface of the contents of the coffee-pot.

When the coffee is ready, one is free to drink it hot and thick, or cold and clear. The gourmets swallow it at once, without waiting; but they who drink it after it has settled, do not omit to gather up the sediment with the little finger and swallow it, that is the sediment, with evident relish. It is asserted positively that coffee thus prepared may be taken without inconvenience ten times in the day, whereas one could not take coffee made in the French fashion five times in the same period. It is a Frenchman who utters this opinion, and we may suppose him to be impartial on the occasion. We should be sorry, however, even with all its reputation for mildness of effect, to see Turkish coffee used a tithe of ten times, by persons with an excitable condition of the nervous system, or who suffer from cardiac irritation.

The writer (M. About) here referred to says that it is a mistake to suppose that the people of the East drink coffee without sugar. In the chief coffee-houses of Athens it is served with sugar, and in the inferior ones of that city it is sweetened in advance.

Both at Smyrna and at Constantinople it was always brought to him too much sweetened.

---

A BRIGHT PICTURE.—Drunkenness, so common in cold climates, is a very rare vice among the Greeks. They are great drinkers,—but it is of water. They make it a point not to pass a fountain without drinking from it, and if they enter a *cabaret*, or wine shop, it is to talk. The Athenian coffee-houses are full of people, at all hours; but there customers do not take strong drinks. They indulge themselves in a cup of coffee, which costs a cent, or in a glass of water, and fire to light their cigarettes, or in a newspaper, or a game of dominoes. These are their indulgences for the day. M. About did not see, in a period of ten years, a man dead drunk in the streets; and he believes that it would be a very easy matter to count all the drunkards in the kingdom of Greece.

What a load of business would be removed from our criminal courts and executive labor from our police force, not to speak of the saving to the public treasury and of the public health, if there were even an approach to this state of sobriety in the several commonwealths of our great Union.

